# A proteomic analysis of the statocyst endolymph in common cuttlefish (*Sepia officinalis*): an assessment of acoustic trauma after exposure to sound

**DOI:** 10.1038/s41598-019-45646-6

**Published:** 2019-06-27

**Authors:** M. Solé, M. Monge, M. André, C. Quero

**Affiliations:** 1 Laboratory of Applied Bioacoustics, Technical University of Catalonia, Barcelona TECH, 08800 Rambla exposició s/n, Vilanova i la Geltrú, Barcelona, Spain; 2 Proteomics Laboratory, Vall d’Hebron Institute of Oncology (VHIO), Edifici Collserola, 08035 Barcelona, Spain; 3 Department of Biological Chemistry and Molecular Modelling, IQAC (CSIC), Jordi Girona 18, 08034 Barcelona, Spain

**Keywords:** Ecology, Environmental impact

## Abstract

Recent studies, both in laboratory and sea conditions, have demonstrated damage after sound exposure in the cephalopod statocyst sensory epithelium, which secretes endolymph protein. Here, the proteomic analysis of the endolymph was performed before and after sound exposure to assess the effects of exposure to low intensity, low frequency sounds on the statocyst endolymph of the Mediterranean common cuttlefish (*Sepia officinalis*), determining changes in the protein composition of the statocyst endolymph immediately and 24 h after sound exposure. Significant differences in protein expression were observed, especially 24 h after exposure. A total of 37 spots were significantly different in exposed specimens, 17 of which were mostly related to stress and cytoskeletal structure. Among the stress proteins eight spots corresponding to eight hemocyanin isoforms were under-expressed possible due to lower oxygen consumption. In addition, cytoskeletal proteins such as tubulin alpha chain and intermediate filament protein were also down-regulated after exposure. Thus, endolymph analysis in the context of acoustic stress allowed us to establish the effects at the proteome level and identify the proteins that are particularly sensitive to this type of trauma.

## Introduction

The introduction of artificial sound sources in the marine environment is known to have potential negative effects on marine organisms. Though marine mammals^1^ and fishes^2^ have been the main focus of research in this area, invertebrates have received increasing attention, with recent data showing that they can be affected by exposure to noise^3–17^. A review of the effects of noise on marine invertebrates was previously published by the authors^18^.

**Figure 1:**
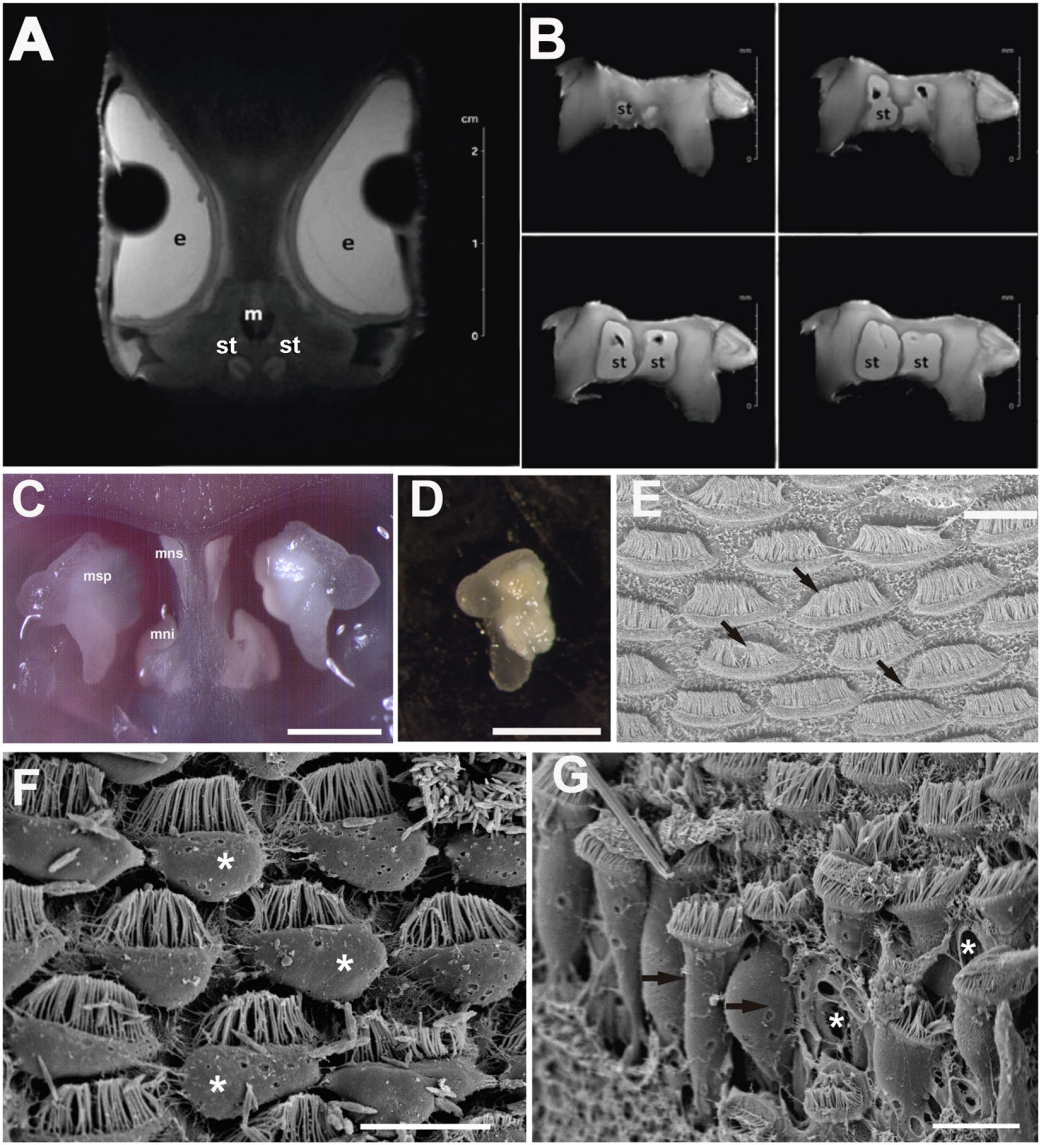
(**A,B**) MRI. *S. officinalis* statocyst location into the cephalic cartilage. (**C**,**D**) LM, photomicrographs of *S. officinalis* statocyst structure. (**E–G**) SEM, S. officinalis macula statica princeps (msp) epithelium. (**A–E**: control animal. (**F,G**): animal sacrificed 24 h after sound exposure). This images comes from a previous analysis^18^. (**A**) Coronal view –anterior section- of cuttlefish (*S. officinalis*) head. (**B**) Four views of cuttlefish cartilage showing the statocyst cavities at different levels. The sections were performed in an antero-posterior direction. The statocyst cavities, corresponding to the white masses in the centre of the images, are filled with endolymph. (**C**) *S. officinalis* inner statocyst structure. Anterior view. The statocyst cavities have been opened transversaly. Each cavity shows the three subunits of the macula-statolith system (msp, mns, mni). (**D**) *S. officinalis* statolith. (**E**) Msp epithelium. The arrangements of the kinociliary groups of the hair cells in regular lines following the epithelium shape are visible. Arrows indicate hair cells’ direction of polarization. (**F**) The apical poles of the hair cells extruded above the epithelium in the statocyst cavity are visible (asterisks). (**G**) Almost all the cell body of hair cells is ejected from a large region of the sensory epithelium (arrows). Some holes on the epithelium due to hair cell inner material extrusion are visible (asterisk). (e: eye, m: mouth, msp: *macula statica princeps*, mns: *macula neglecta superior*, mni: *macula neglecta inferior*, st: statocyst). Scale bars: A, B = 2 cm. C, D = 2,5 mm. E, F, G = 10 µm.

Cephalopods are short-lived marine invertebrates that exhibit sensitivity to environmental change and stressors^19^, with complex behavioural patterns^20^, and play a significant role as both prey and predator^21–24^. These characteristics allow responses to changes in the marine environment^25–27^, including underwater noise effects, to be assessed. All cephalopods have a pair of statocysts located within the cephalic cartilage (Fig. 1). The importance of the statocyst as a fundamental system for the regulation of vital cephalopod behaviour, including locomotion, posture, balance, and movement in the water column, is undeniable^28,29^. The statocysts are sophisticated balloon-shaped bodies filled with endolymph and contain sensory hair cells on the inside wall of the inner sac. Statocysts are grouped into two main areas of sensory epithelium: the *macula*-statolith system and *crista*-*cupula* system (Fig. 1C–E)^28–31^. The *macula*-statolith system controls changes in position according to gravity and linear acceleration, whereas the *crista*-*cupula* system determines changes in the angular acceleration. Several studies have been performed on the structural and biochemical composition of cephalopod statoliths (Fig. 1D), calcified biomineral structures composed of calcium carbonate crystallized as aragonite with a small percentage of organic material (proteins)^32^. However, very few initiatives have focused on characterizing the organic matrix proteins of statolith^33^ or quantifying the protein concentration of statolith and statocyst endolymph^34^. These proteins play an important role in the statolith formation through its deposit together with aragonite component, and therefore they are directly associated to all functions of the statolith-statocyst complex. Modification of the statocyst endolymph protein content after exposure to sound could induce improper physiological functioning of this organ, which would result in defective regulation of these vital functions for survival*. Sepia officinalis* Linnaeus 1758 is a commercial demersal species that lives on muddy or sand platforms covered by seagrass and algae, down to a depth of 200 m, in the Mediterranean Sea and Northwest Atlantic Ocean. This species performs seasonal inshore spawning and offshore migrations to feed in cold waters^35^. Amongst other consequences, if the endolymph protein content is modified by sound exposure, cuttlefish may not be able to perform the inshore migration in order to breed and lay eggs on the seabed substrate or the offshore migrations to colder waters to feed.

Recent analyses in laboratory^18,36–38^ and offshore conditions^39^ by scanning electron microscopy (SEM) and transmission electron microscopy (TEM) have revealed damage in the inner sensory epithelium of statocysts in cephalopods exposed to sound (Fig. 1E–G) and lobsters^40^. After exposure, the kinocilia on hair cells were either missing, or bent or flaccid. A number of hair cells had protruding apical poles (Fig. 1F) and ruptured lateral plasma membranes, likely resulting from the extrusion of cytoplasmic material. The whole hair cell body was also ejected from the sensory epithelium (Fig. 1G). This damage resulting from exposure to artificial noise directly affected the functionality and sensitivity of the cephalopod statocysts. Given that the statocyst sensory epithelia are responsible of the secretion of statocyst endolymph protein, here we investigate if noise exposure induces proteomic changes in the endolimph by exposing N Mediterranean common cuttlefish, *S. officinalis*, to the same low frequency sound regime that was observed to cause physiological damage to the sensory epithelia in previous studies. A comparative analysis of the cuttlefish proteome at different times after sound exposure was performed to detect differences in protein abundance with the objective of determining proteins more sensitive to acoustic related stress.

## Results

### Protein content

We measured (mean ± SD) 11.7 ± 6.0, 17 ± 6.6, and 13.1 ± 2.0 μg protein per statocyst in non-exposure controls (C; N = 24), 0 h after exposure (T0; N = 32), and 24 h after exposure (T24; N = 24), respectively (Fig. S1). No significant differences were found in the overall protein concentration between treated and untreated individuals (F = 0.209, P = 0.816; one-way ANOVA). All samples were processed at the same time of day (14:00–15:00) and the protein content varied from 0.8 to 1.13 μg μl^−1^ endolymph (Fig. S1).

### Differences in two-dimensional difference gel electrophoresis (2D-DIGE)

**Figure 2:**
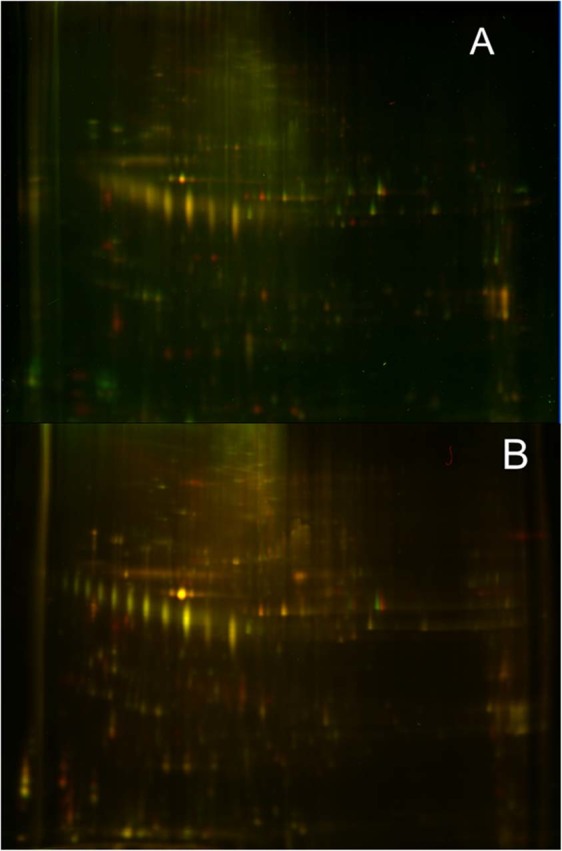
Representative two-dimensional differential gel electrophoresis (2D-DIGE) fluorescent image of *Sepia officinalis* endolymph labeled with CyDyes corresponding to the overlapping Cy3 Dye (green spots) and Cy5 Dye (red spots). Merge spots appeared in yellow. First dimension was pH 3 to 10 linear IPG gel and second dimension was a range of 15 to 200 KDa in a 12.5% gel. Comparison of proteomes of endolymph sample of (**A**) Control (Cy5-labeled) vs 24 h (Cy3-labeled) and (**B**) 0 h (Cy3-labeled) vs 24 h (Cy5-labeled) after sound exposure.

**Figure 3:**
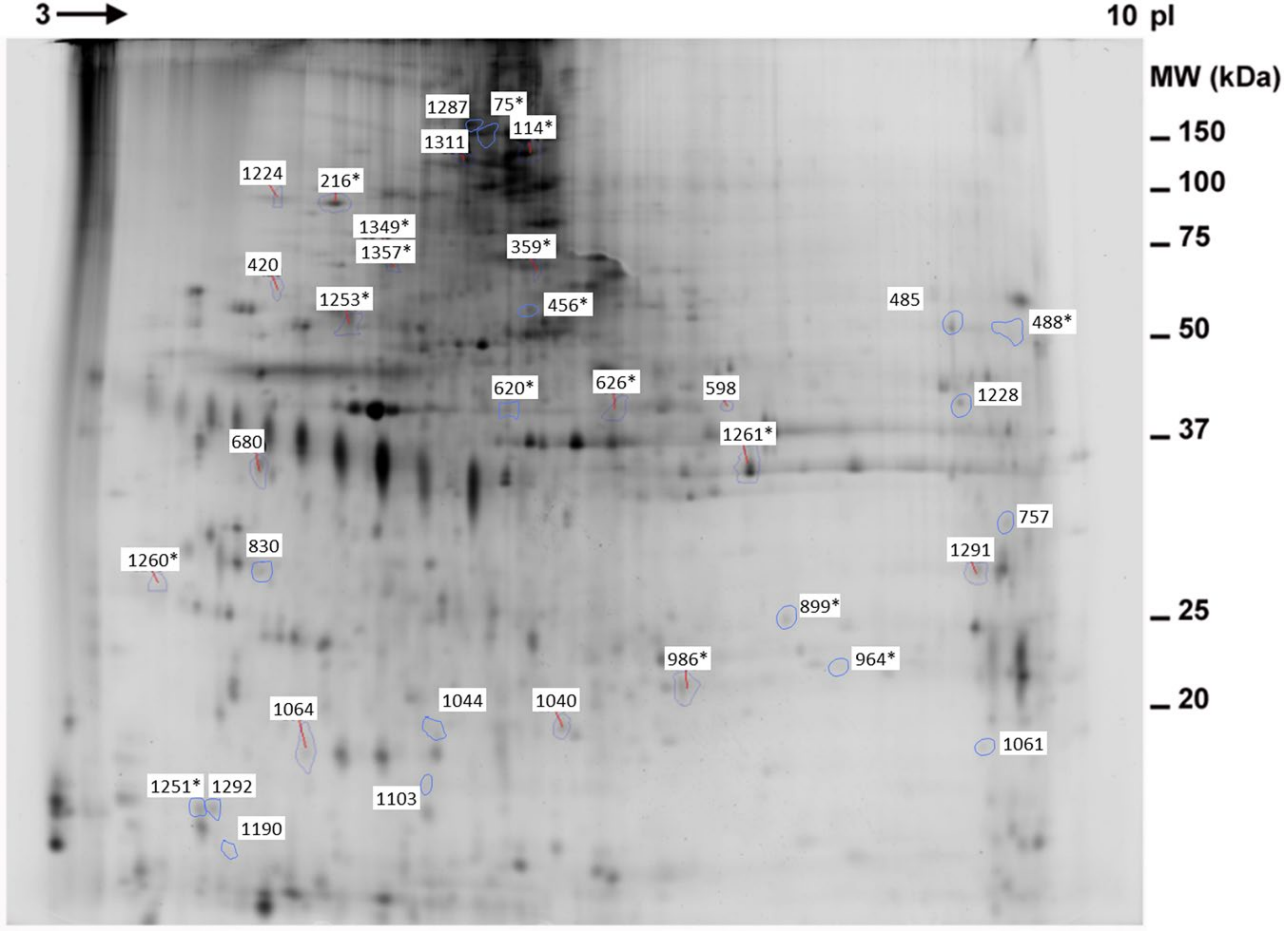
Two-dimensional electrophoresis map of *Sepia officinalis* endolymph. Soluble proteins were separated on linear IPG-strips (pH 3–10) followed by 12.5% SDS-PAGE and then flamingo stained. Numbered spots indicate that there are differences among treatments within a significant confidence level (p < 0.05) and those protein spots that have been identified are indicated with an asterisk next to the number.

**Figure 4:**
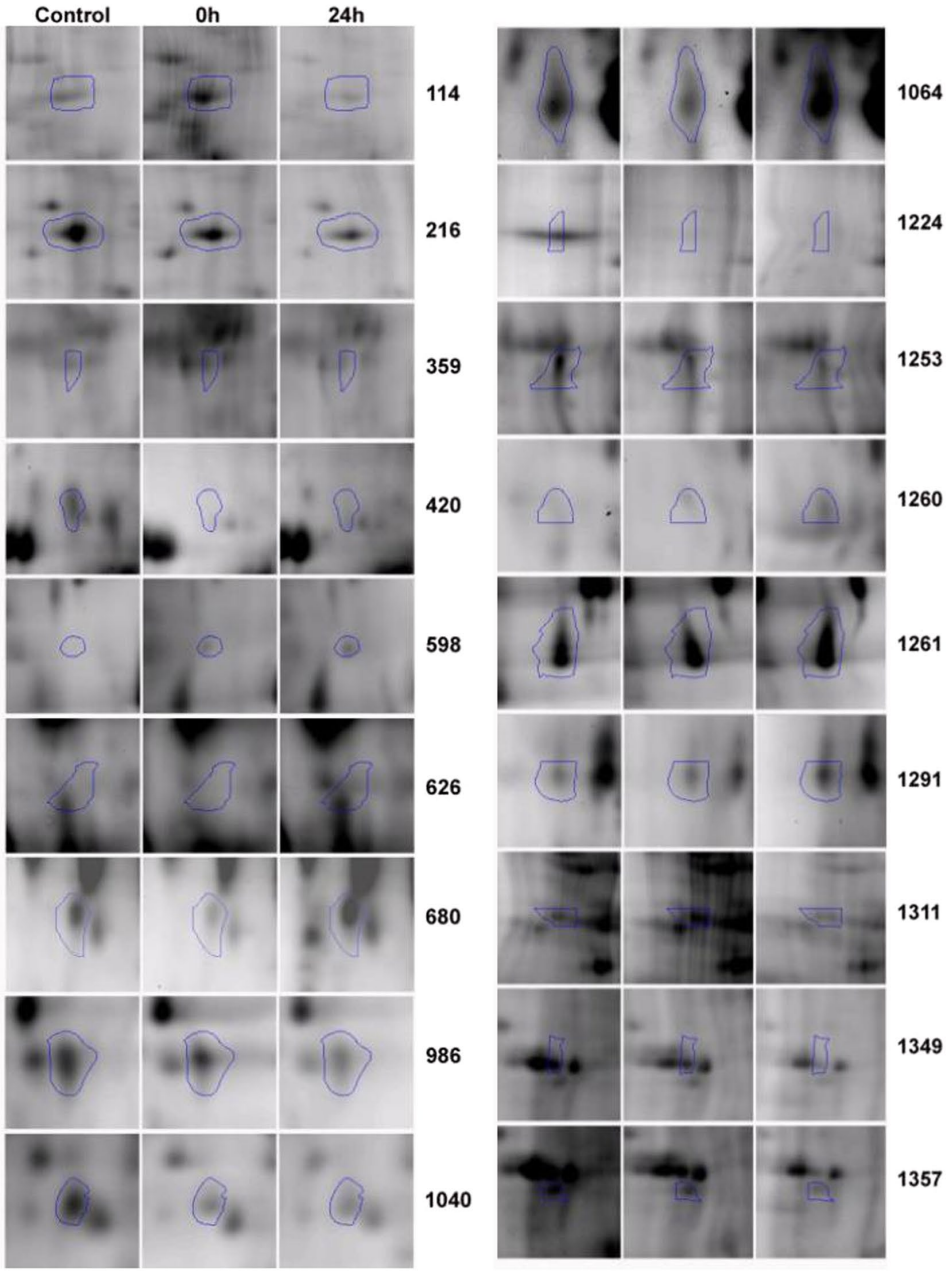
Selected 2D-PAGE gel areas related to *Sepia officinalis* endolymph proteins differentially expressed according to treatments (C, 0 h and 24 h). Full length image is shown in Fig. 3.

**Figure 5:**
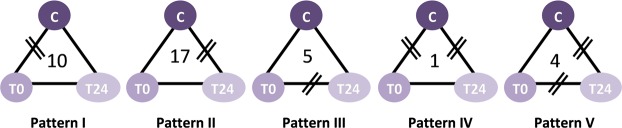
Distribution of differentially expressed protein spots. The 2-DE gel analysis revealed 37 differentially expressed spots that were compared between every two of the three samples and subsequently distributed into five patterns. Two bars on the union line of the treatments indicate significant differences between treatments (For example: pattern I, ten spots differentially expressed between control (C) and 0 h treated; IV, one spot differentially expressed between control (C) and 0 h treated (T0) as well as sample control and 24 h treated (T24), but not sample T0 and T24, …)).

Protein extracts from the endolymph of untreated and treated individuals were analysed by 2D-DIGE, allowing us to compare three protein samples simultaneously on the same gel. The proteomic profiles show an average of approximately 900 spots (Fig. 2). A comparison of the locations and volumes associated with each spot revealed that the majority of proteins remained unchanged between C and T0 (Figs 3 and 4). The variation in spot intensities was much clearer between C and T24. The expression levels of 37 of the matched proteins were significantly different between individuals at different times (Figs 2 and 3, Table S2). These differentially expressed proteins could be distributed into five patterns (Fig. 5): pattern I, 10 proteins differentially expressed between C and T0; pattern II, 17 proteins differentially expressed between C and T24; pattern III, five proteins differentially expressed between T0 and T24; pattern IV, one protein differentially expressed between C and T0 and C and T24, but not T0 and T24; and pattern V, four proteins differentially expressed between C and T24 and T0 and T24, but not C and T0. The most important differences were found between C and T24. A total of 22 spots (17 in pattern II, 1 in pattern IV, and 4 in pattern V) were expressed differentially in both treatments. Four were also differentially expressed between T0 and T24. Only 11 proteins were differentially expressed just after treatment (T0).

### Identification

Protein characterization was carried out by matrix assisted laser desorption/ionization-time of flight mass spectrometry (MALDI-TOF MS). Mass fingerprints were compared to those of known proteins from several protein databases (Swiss-Prot and non-redundant NCBI database). Twenty one of the 37 differential spots were identified by peptide mass fingerprinting and liquid chromatography-electrospray ionization (LC-ESI) (Table 1). Few proteins were identified due to the scarcity of molluscan sequences in public databases and the absence of an assembled cephalopod genome. Most of the spots identified in the 2-D gels corresponded to a single protein. However, we identified proteins that were not separated; for example, spots 456 and 1357 contained two proteins each. Furthermore, some proteins appeared as several spots on the 2-D gels, corresponding to isoforms of the same protein. These protein isoforms could be a consequence of variably spliced forms of the same gene product, N- and C-terminal truncations, or post-translational modifications.

**Table 1:** *Sepia officinalis* proteins with differential expression between different treatments (Control (C), 0 h (T0) and 24 h treated (T24)).

	#	Identification^a^	Increase/decrease^b^			NCBInr^c^	p*I*^d^		Mr^d^ (kDa)	
			C/T0	C/T24	T0/T24		Theoretical	Observed	Theoretical	Observed
Hemocyanin	75	hemocyanin subunit 2 [*Sepia officinalis*]	=	=	−	gi|88657469	5.85	5.68	385	148
	114	hemocyanin subunit 2 [*Sepia officinalis*]	=	−	=	gi|88657469	5.85	5.95	385	131
	280	hemocyanin subunit 2 [*Sepia officinalis*]	+	=	−	gi|88657469	5.85	5.60	385	83
	359	hemocyanin subunit 2 [*Sepia officinalis*]	=	−	=	gi|88657469	5.85	6.03	385	71
	626	hemocyanin subunit 2 [*Sepia officinalis*]	=	+	=	gi|88657469	5.85	6.55	385	43
	986	hemocyanin, units G and H	=	−	=	gi|21264302	6.31	7.05	64	21
	456	hemocyanin subunit 2 [*Sepia officinalis*]	=	=	−	gi|88657469	5.85	6.01	385	59
	1357	hemocyanin subunit 2 [*Sepia officinalis*]	=	−	=	gi|88657469	5.85	5.18	385	72
Elongation	488	elongation factor-1 alpha [*Quilaphoetosus monachus*]	=	=	+	gi|55420740	8.53	8.93	46	54
	620	elongation factor 1-alpha *[Xyleborus biuncus*]	=	−	=	gi|21105549	8.74	5.83	31	43
	869	elongation factor 1-a [*Hunterella nodulosa*]	=	=	+	gi|6467315	6.67	5.26	29	26
	1251	elongation factor-1 alpha [*Chloreuptychia arnaca*]	=	+	=	gi|55420670	8.53	4.21	45	<20
	964	elongation factor-1 alpha [*Maculinea arion*]	−	=	=	gi|56123383	8.23	8.06	38	22
Others	216	heat shock protein 90 [*Dendronephthya klunzingeri*]	=	−	=	gi|14041148	4.80	4.92	84	95
	456	tubulin alpha chain	=	=	−	gi|48428558	5.00	6.01	51	59
	899	thioredoxin peroxidase BgTPx [*Biomphalaria glabrata*]	−	=	=	gi|13488586	8.36	7.72	25	25
	1253	tubulin alpha chain	=	−	=	gi|48428558	5.00	4.99	51	59
	1260	proteasome alpha subunit	=	+	+	gi|31211921	4.73	4.00	27	28
	1261	glyceraldehyde-3-phosphate dehydrogenase 1	=	+	=	gi|6016070	6.97	7.47	35	37
	1349	70 kDa neurofilament protein	=	−	=	gi|266617	5.31	5.17	71	73
	1357	intermediate filament protein [*Nototodarus sloanii*]	=	−	=	gi|159852	5.29	5.18	70	72

^a^Proteins identified after in-gel digestion with trypsin and MALDI-TOF MS and LC-ESI analysis (# corresponds to numbers in Figs 3 and 4); ^b^Significant differential expression (p < 0.05) between treatments which were maintained (=), increased (+) or decreased (−) 1.4-fold; ^c^Mowse score > 67; ^d^Sometimes theoretical and experimental p*I* and Mr differ. Such differences could result from the detection of a dimer or a fragment of the protein, or posttranslational modifications. (More details in Table S1).

Some of the differentially expressed proteins were identified and related to stress reaction (e.g., hemocyanin, elongation factor 1-alpha [EF-1a], thioredoxin peroxidase, proteasome alpha subunit, and heat shock protein), and others are known to have structural functions (e.g., tubulin alpha chain, 70 kDa neurofilament protein, and intermediate filament protein). Seven of the identified spots (75, 114, 280, 359, 456, 626, 986, and 1357) corresponded to the protein hemocyanin of *S. officinalis*. The expression of these proteins present in the endolymph decreased in abundance 24 h after exposure compared to control specimens or immediately after exposure (Table 1). Two of the differential spots increased in volume. Another protein identified in multiples spots was EF-1a. This protein remained almost unchanged at T0, but was up-regulated at T24 compared to C (spot 1251) or T0 (spots 488 and 869). Two of the identified isoforms were down-regulated (spots 620 and 964).

Four more of the identified proteins were related to stress situations; two were down-regulated after exposure (heat shock protein [spot 216] and thioredoxin peroxidase BgTPx [spot 899]) and two were up-regulated (proteasome alpha subunit [spot 1260] and glyceraldehyde-3-phosphate dehydrogenase 1 [spot 1261]). Expression of the 90-kDa heat shock protein (HSP90) significantly decreased at T24 compared to C, but no difference was found between C and T0. In contrast, thioredoxin peroxidase decreased at T0 and recovered by T24. In the case of the two up-regulated proteins, the changes were observed at T24.

The group of proteins related to structural functions (i.e., tubulin alpha chain [spots 456 and 1253], 70 kDa neurofilament protein [spot 1349], and intermediate filament protein [spot 1349]) had decreased expression 24 h hours after exposure.

## Discussion

Although a previous work showed proteomic changes after sound exposure in birds^41^, these techniques were for the first time used here in a marine species. Previous studies^17,34–36^ showed the effects of sound exposure on statocyst sensory epithelia. The hair cells of the statocyst sensory epithelia perform important roles, secreting the macromolecules that constitute the organic matrix of the statolith, providing the necessary ionic environment for controlled mineralization and exerting spatiotemporal control over these events. The fact that noise exposure affects the hair cells of the statocyst sensory epithelia, combined with the observations in fishes that the saccular epithelial cells in the inner ear are responsible for endolymph protein secretion^42^, suggests that the endolymph proteome is affected by this external stimulus. Here we have recorded delayed changes in the expression of stress proteins in response to the same sound exposure that was observed to damage the sensory epithelium, providing a mechanistic explanation for sound impacts relating morphological and biochemical effects.

The protein content in controls and exposed animals was compared at the same time of day. Only slight differences were observed in the protein concentrations, probably due to intrinsic variability between individuals. The quantity of protein detected in the statocyst was similar to that observed by Bettencourt and Guerra^34^, who found that the protein concentration in the endolymph of *S. officinalis* changed from 1.8 μg μl^−1^ in the morning to 0.7 μg μl^−1^ in the evening.

After comparing the proteome maps of controls and the two experimental groups, the most important differences were between C and T24, with fewer differentially expressed proteins just after exposure (T0). However, as important damage in the statocyst sensory epithelia was found just after exposure to noise^18,38^, these results show that changes in proteome expression are expressed at a later stage than structural changes. Protein abundance changes as a response after stress conditions could be regulated not only at the transcriptome level but also at the level of translation and protein degradation, then it could take place before the transcriptome response and it could last for a longer period^43^. Because we did not perform our analysis at different intervals between 0 h and 24 h, we cannot directly compare the expression changes along this period but we observed a delay between the stress induction and the protein response and these changes lasted for at least 24 h after treatment. Nevertheless, the changes in protein expression observed 24 h after exposure could have appeared at any moment between 0 and 24 h, following the sequential structural changes on the epithelia, which was observed to start just after sound exposure^18^, as reported by other authors in acoustically over-stimulated avian inner ears^44^.

Some proteomic studies have been performed in other marine animals to check changes at the proteome level in response to external environmental events, such as temperature^45–47^, osmolality^48^, and oxygen concentration^49^. These studies showed that protein changes occurred in response to a novel environment with the purpose of maintaining basic cellular functions and limiting cellular damage. The present results show different levels of changes in the cuttlefish proteome as a direct consequence of sound exposure, mainly related to stress and structural functions.

Among the stress-related proteins that were differentially expressed, hemocyanin was identified in eight of the differential spots. This main protein in the hemolymph of many invertebrates, which is responsible for oxygen transport, is an extracellular respiratory pigment (3.9 MDa) composed of 10 polypeptide chains, each containing seven or eight functional subunits of 75 kDa^50^. The spots that were identified as hemocyanin at various p*I* and MW have remarkably lower apparent masses than the computed mass for the whole protein, indicating that these multiple spots contained one or two subunits or only a fragment of the identified protein. We observed few changes in expression just after noise exposure, but 24 h after treatment most hemocyanin fragments were down-regulated, and only one fragment of hemocyanin was up-regulated. Proteomic studies in the hemocytes of shrimp (*Penaeus vannamei*) after Taura Syndrome viral infection have shown that changes in the expression of hemocyanin C- and N-terminal fragments are related to an increase in antiviral activity and oxygen transport, respectively^51^. In addition, the C-terminal hemocyanin fragments had more acidic p*I* than the N-terminal fragments. In our case, the down-regulated spots identified as hemocyanin corresponded to fragments localized in a pH range of 5.8 to 7.05 (see Fig. 3 and Table 1). In these spots, the decrease in expression after exposure to sound is probably related to a reduction in oxygen consumption, and could be due to stress evoked by noise exposure or any other mechanisms that may have occurred in the statocyst to compensate for the physiological changes occurring in the hemolymph.

Other down-regulated stress proteins were identified as HSP90 and the thioredoxin peroxidase BgTPx. Heat shock proteins are ubiquitous in nature; some are ephemerally synthesized in response to metabolic and environmental stress by acting as molecular chaperones, helping to refold the misfolded proteins^52^, whereas others are constitutively expressed in unstressed cells and contribute to normal processes. Among the different functions described for HSP90, special mention is given to the facilitation of cytoskeletal rearrangement and association of the protein with tubulin monomers and microtubules^53,54^. In some studies, HSP90 levels were up-regulated when animals were under stress. However, in other studies, the expression of HSP90 was down-regulated^55^. In the present study, we observed a clear decrease in the expression of HSP90 that was likely related to its interaction with cytoskeletal proteins. Thioredoxin peroxidase was also down-regulated under stress conditions. This protein plays an important role as an antioxidant enzyme^56,57^ removing various reactive oxygen species (ROS) produced by free radical reactions^58^. For example a decrease in thioredoxin peroxidase causes an increase on ROS levels, triggering apoptosis in the mammal inner ear organ of Corti^59^, the extrusion process of the hair cells in the avian inner ear^60^, and in the sensory epithelia of cephalopods after sound exposure.

Other groups of proteins that changed with sound exposure represent the cytoskeleton. Sound exposure leads to down-regulation of cytoskeletal proteins, including tubulin alpha chain, an intermediate filament protein, and a 70-kDa neurofilament protein. Tubulin alpha chain is a constitutive microtubule protein and one of the main structural components of cilia in eukaryotic cells that plays a key role in flagellar motility^61^. The intermediate filament is a primary component of the cytoskeleton, providing mechanical strength to cells and tissues and creating cell cohesion, preventing the acute fracture of epithelial cell sheets under tension and helping stabilize the extended axons of nerve cells^62^. Finally, the 70-kDa neurofilament protein is one of the major components of the axonal intermediate filaments of all vertebrate and many invertebrate neurons. The decreased expression of these proteins is consistent with destruction of the microtubular structures of the statocyst sensory epithelium (cilia, microvilli, centrioles) observed previously as a consequence of sound exposure^18,37–39^. In addition to primary hair cells, cephalopod statocysts have secondary sensory hair cells, first-order afferent neurons, and efferent nerve fibres. The neurofilaments present in all of these elements would be affected by sound exposure in the same way as cytoskeletal elements of the mammalian organ of Corti^63–65^ and in the avian auditory epithelium^44^. The downregulation of these structural proteins is consistent with hypertrophy of the nervous afferent system and nerve endoplasmic reticulum damage, vacuolization, or complete degeneration as reported in our previous study^18^. The cytostructural damage provokes a breakdown of synaptic function, which can be enhanced by the leak of potassium-rich fluid. Similar lesions have been described in the mammalian inner ear^66^ and the cerebral cortex and hippocampus after sound exposure^67^. As some signs of recovery have been observed in the sensory epithelia at the structural level by imaging 48 h after sound exposure^18^, we hypothesized that an increase in the expression of these cytoskeletal proteins could be triggered in animals sacrificed after this period. Therefore, the effect on protein expression needs to be determined after a longer period of time.

The remaining spots that were identified are involved in metabolic processes, such as glycolysis, proteolysis, and protein synthesis, and all of them were up-regulated after sound exposure. Several spots corresponded to EF-1a. This protein has been extensively associated with peptide chain elongation in protein biosynthesis^68^, but it may also be involved in the facilitation of substrate mobilization to ubiquitin proteasome pathways and protein degradation^68,69^ or the cell apoptotic process in response to oxidative stress^70,71^. In the present study, up-regulation of three spots identified as EF-1a could be involved in the response to sound exposure by increasing specific protein synthesis and the degradation of other specific proteins as described in other studies^45,47^.

Up-regulation of the proteasome alpha subunit was observed 24 h after exposure. Proteasomes play many roles in the cell, including the removal of abnormal and misfolded proteins from the cell. Proteasomes are also involved in the cell’s stress response, regulation of the cell cycle, and cellular differentiation. The increase in proteasome activity could be evidence of dysfunction of the sensory epithelia and in the processes that occur in the statocyst as a consequence of sound exposure.

All of the proteins that have changed their expression are implicated in essential mechanisms for the survival and reproduction of the species. When organisms are exposed to damaging stress conditions (e.g., noise), major alterations in gene expression and cell physiology could occur to compensate for the damage. In our study, these changes included the down-regulation of proteins implicated in oxygen transport, posttranslational modification, and signal transduction, and the up-regulation of other proteins implicated in folding and unfolding and posttranslational modifications.

Despite protein databases for cephalopods, and specifically *S. officinalis*, being scarce, 10 proteins were successfully identified by a homology search using all sequence data available in public databases. The number of differential spots could only be partially identified, probably because they are not described or because of the considerable variations in trypsin hydrolytic peptide masses for homologous proteins. More studies should be performed to identify these differential proteins, such as through preparative gels and other analytical methods (MS-MS).

## Conclusion

This study reports the first analysis of the effects of low frequency sound exposure on the cephalopod endolymph proteome. Our experiments with cuttlefishes provide evidence of different levels of expression of proteins in the endolymph after exposure to sounds. These alterations not only affect the sensory epithelia as this was demonstrated in previous studies^18^, but also the statolith structure. As the statolith is formed by daily calcium and protein deposition, modifications in the amount of protein may induce structural changes in the statolith. Consequently, the physiology and vital functions of the *S. officinalis* statocyst could directly affect the exposed individual’s sensory information. More work is required to understand those proteomic changes and their possible persistence over time and their long-term effects on individuals and populations.

## Material and Methods

### Cuttlefish individuals

As a preliminary step before starting with the noise exposure experiment, a set of 30 individuals of *S. officinalis* were kept in the tanks for several weeks to analyse their adaptation to captivity. These animals were normally swimming, eating, mating, laying eggs and behaving typically over the entire observation period. These specimens were not further used for the exposure experiments. After confirming the correct adaptation of the cuttlefishes to our experimental tanks, we started the exposure experiments with fresh newly-caught specimens. Eighty adult *S. officinalis* individuals (mantle length 11–18 cm) were caught by local fishermen following the same protocol as in previous controlled exposure experiments^18^. Animals were collected from the Catalan coast (NW Mediterranean Sea) using basket traps and transferred within 1 h after capture to a closed system of recirculating natural seawater (at 18–20 °C, salinity 35‰, and natural oxygen pressure under regular monitoring). This system consisted of two mechanically filtered fiberglass-reinforced plastic tanks (2000 L capacity) that were connected to each other (Laboratory of Applied Bioacoustics – UPC, Vilanova i la Geltrú fishing harbour). This setup included a physicochemical self-filtration system with activated carbon and sand driven by a circulation pump. Individuals were supplied *ad libitum* with live crab (*Carcinus maenas*) as a food source ad libitum and maintained in the tank system until exposure.

### Ethics issue

The experimental protocol strictly followed the necessary precautions to comply with current ethical and welfare considerations when dealing with cephalopods in scientific experimentation^72–74^. The animal facility was approved by the Ministry of Agriculture, Livestock Fisheries and Food -Generalitat de Catalunya  (approval code B9900085). This process was also carefully analysed and approved (ref. number CEA/10301) by the Animal Welfare Committee on Animal Experimentation of the Ministry of Agriculture, Livestock, Fisheries and Food - Generalitat de Catalunya and by the Ethical Committee for Scientific Research of the Technical University of Catalonia (CÈEA code 2018-01).

### Sound exposure protocol

The sequential controlled exposure experiment (CEE) was performed as described previously^18,38^. Cephalopods (all individuals) were maintained in the tank system until exposure, which consisted of 50–400 Hz sinusoidal wave sweeps with 100% duty cycle and a one-second sweep period for 2 h. The sweep was produced and amplified through an in-air loudspeaker while the level received was measured by a calibrated B&K 8106 hydrophone. Sound pressure level measurements were made starting from the moment that the sound field in the tank had stabilized (including any acoustic effects produced by the tank or the room). The exposure was continuously repeating sweep for a period of two hours. The sweep consisted of a linearly modulated sinusoidal wave with frequencies running from 50 Hz to 400 Hz in 1 second. The speaker response was not uniform over the frequencies covered by the constant amplitude sweep. The sound pressure level (SPL) of a 1 second sweep was 157 dB re 1 μPa^2^ with peak levels up to 175 dB re 1 μPa^2^. The sound exposure level (SEL) for the 2 hour experiment was 196 dB re 1 μPa^2^s. These are representative levels measured in the centre of the tank where the cuttlefish where remaining most of the time during the experiments.

In a previous work, both particle motion and sound pressure levels^36^ were modelled. These confirmed the heterogeneity of the measurements due to the tank experimental conditions. In another offshore experiments reproducing the present CEE, it was possible to determine that animals were exposed at levels ranging from 139 to 142 dB re 1 μPa^2^ and from 139 to 141 dB re 1 μPa^2^, at 1/3 octave bands centred at 315 Hz and 400 Hz, respectively^39^.

The choice of the acoustic parameters to be used in the experiments was based on both the sensitivity to sound of the species as well as because most human activities at sea commonly introduce these noises in areas where cuttlefishes are found. The ecological relevance of this study has been described in previous publications^18,36–39,75,76^, especially when the laboratory findings were confirmed by results obtained at sea in natural conditions^39^.

After the exposure, some individuals were immediately sacrificed (T0; N = 32). Others were placed in a tank for 24 h before being sacrificed (T24, N = 24). Some of the animals were used as controls (C; N = 24) and kept in the same conditions as the experimental animals in an independent tank and sacrificed in the same sequential process but without exposure to noise. Tank for control animals was located in a separate location, acoustically isolated from exposed tanks.

### Removal of statocysts and extraction of endolymph

Isolated head preparations obtained by decapitation were used in all experiments. Statocysts with surrounding cartilage were removed and 10–40 μl of endolymph extracted from every statocyst with a microsyringe. Given the same sound regime was applied than in previous experiments we assume that the epithelium was damaged. The use of micro syringes can affect the sensory epithelium. For this reason in the present work the statocysts inner epithelia were not analysed on animals that were used for proteomic analysis.

The endolymph was then frozen at −70 °C until required for protein analysis. The same protocol was applied to both controls and experimental groups. Three different biological samples of 100–150 μl were obtained from each treatment, corresponding to four to six animals per sample depending on the amount of hemolymph extracted from each statocyst. All of the animals were sacrificed and endolymph extracted at 14:00–15:00 h to minimize daily variations in endolymph protein concentrations. One-way ANOVA test was conducted for comparison of mean values from the three treatments

### Two dimensional-difference gel electrophoresis

Two-dimensional difference gel electrophoresis (2D-DIGE) is a proteomic methodology that combines traditional 2D gel electrophoresis with fluorescent protein tags. Separation in the first dimension is by native charge using isoelectric focusing and in the second dimension is by molecular weight. The main feature of this technique is the use of fluorescent dyes to label protein samples so that multiple samples can be run on the same gel with a very high sensitivity and changes in protein expression can be quantified^77,78^.

Three samples of four to six animals were used for each treatment. All samples were adjusted to 200 μl with lysis buffer (7 M urea, 2 M thiourea, 4% w/v CHAPS, 2% w/v dithiothreitol [DTT], 30 mM Tris-base) before sonication (three pulses of 2 sec at 10-sec intervals). Because the high levels of salt present in the endolymph could interfere with focusing the proteins, the salt content was removed from the samples (PlusOne 2-DE clean up kit, Amersham Biosciences, Little Chalfont, Bucks, UK) and resuspended in lysis buffer.

Protein content was quantified (RC DC Protein Assay, Bio-Rad, Hercules, CA) before labelling with cyanine dyes (Cy3 or Cy5) by the addition of 400 pmol of Cy dye in 1 μl of anhydrous N,N-dimethylformamide per 30 μg of protein. A pool consisting of equal amounts of each sample analysed was prepared for use as an internal standard for quantitative comparisons^79^. To avoid any possible bias introduced by labelling efficiency, half of the samples from each group were labelled with Cy3 dye and the other half with Cy5 dye. After 30 min of incubation on ice in the dark, the reaction was quenched by the addition of 1 μl of 10 mM lysine and incubated for 10 min. The samples were finally combined according to the experimental design (Table S1; 30 μg of protein per Cy dye per gel) and diluted 2-fold with isoelectric focusing sample buffer (7 M urea, 2 M thiourea, 4% w/v CHAPS, 2% w/v DTT, 2% v/v pharmalytes pH 3–10, 0.002% bromophenol blue). 2D-DIGE was performed using GE-Healthcare reagents and equipment. Isoelectric focusing was performed on immobilized pH gradient (IPG) strips (24 cm; linear gradient pH 3–10) using an Ettan IPGphor system (Amersham Biosciences). Samples were applied via anodic cup loading on IPG-strips previously incubated overnight in 450 μl of rehydration buffer (7 M urea, 2 M thiourea, 4% w/v CHAPS, 1% v/v pharmalytes at pH 3–10 [GE Healthcare], 100 mM DeStreak [GE Healthcare], 0.002% bromophenol blue). After focusing for a total of 67 kVh, IPG-strips were equilibrated first for 15 min in 6 ml of reducing solution (6 M urea, 100 mM Tris-HCl, pH 8, 30% v/v glycerol, 2% w/v SDS, 5 mg ml^−1^ DTT, 0.002% bromophenol blue), and then in 6 ml of alkylating solution (6 M urea, 100 mM Tris-HCl [pH 8], 30% v/v glycerol, 2% w/v SDS, 22.5 mg ml^−1^ iodoacetamide, 0.002% bromophenol blue) for 15 min on a rocking platform. Second-dimension electrophoresis was run by overlaying the IPG-strips on 12.5% isocratic Laemmli gels (24.6 × 20 cm) on an EttanDALTsix system (Amersham Biosciences). IPG-strips were sealed to the gel with 0.5% agarose in reducing solution without DTT. A piece of filter paper (0.2 × 0.5 cm) containing 1 µl of molecular weight marker (Sigma-Aldrich) was sealed at the basic end of the focusing gel. Gels were run at 20 °C at a constant power of 2.5 W/gel for 30 min, followed by 17 W/gel until the bromophenol blue tracking front reached the end of the gel.

Fluorescence images were acquired on a Typhoon 9400 scanner (GE Healthcare). Cy2, Cy3, and Cy5 images were scanned at excitation/emission wavelengths of 488/520 nm, 532/580, and 633/670 nm, respectively, at a resolution of 100 μm.

### Differential image analysis

Gels images were processed with Progenesis SameSpots v 4.0 (from Nonlinear Dynamics Ltd. Newcastle, UK) in order to find differences between different treatments. Quantification of spot intensity data was performed by the differential in-gel analysis (DIA) module of DeCyder software: all spots from each gel were detected and normalized volume ratios for each protein were calculated by using the individual signal of pooled-sample Cy2-labeled as an internal standard. Protein spot variation was considered significant if the normalized spot volume showed at least ± 1.4 fold change and a p-value < 0.05 (ANOVA). Protein spots that satisfy these parameters were marked as protein of interest. Internal calibration of the 2D-DIGE gel images with regard to pI and molecular weight was carried out with SameSpots built-in tools.

Gels were post-stained using the noncovalent fluorescent stain Flamingo (BioRad, Hercules, CA). Fluorescence images were then matched to the DIGE analysis. Protein spots of interest were excised from the gel using an automated Spot Picker (GE Healthcare). In-gel trypsin digestion was performed using autolysis stabilized trypsin (Promega) and the tryptic digests purified using plates (Millipore).

### Protein identification

Tryptic digests from excised spots were analysed by MALDI-TOF MS on an Ultraflex TOF-TOF Instrument (Bruker, Bremen, Germany). Samples were prepared using α-Cyano-4-hydroxycinnamic acid as matrix on Prespotted AnchorChip targets (Bruker). Calibration was performed in the external mode using a peptide calibration standard kit (Bruker). The spectra were processed using Flex Analysis 3.0 (Bruker). Peak lists were generated using the ions in the m/z 800–4000 region, with a signal-to-noise threshold > 3. The SNAP algorithm included in the software was used to select monoisotopic peaks from the isotopic distributions. After removing the m/z values corresponding to usually observed matrix cluster ions, an internal statistical calibration was applied. Peaks corresponding to frequently seen keratin and trypsin autolysis peptides were then removed. The resulting final peak list was used to identify the proteins by peptide mass fingerprint (PMF). Mascot ver. 2.2 (Matrix Science Ltd., London UK) was used to search against the NCBI non-redundant database, limiting the search to “other metazoan” (289,245 sequences). The search parameters were: trypsin cleavages excluding N-terminal to P, one or two missed cleavages allowed, cysteine carbamidomethylation set as fixed modification, methionine oxidation as variable modification, mass tolerance less than 50 ppm, and monoisotopic mass values. The criterion for positive identification was a significant Mascot probability score (>67, p < 0.05). Alternatively, proteins were identified by ion trap mass spectrometry^80^. The mass spectrometry proteomics data have been deposited to the ProteomeXchange Consortium via the PRIDE partner repository with the dataset identifier PXD013694^81^.

## Supplementary information

A proteomic analysis of the statocyst endolymph in common cuttlefish (Sepia officinalis): an assessment of acoustic trauma after exposure to sound

## References

[1] Richardson, W. J., Greene, J. C. R., Malme, C. I. & Thomson, D. H. *Marine mammals and noise* (Academic Press, 1995).

[2] Popper AN, Hastings MC (2009). The effects of human‐generated sound on fish. Integr Zool.

[3] Aguilar de Soto, N. In *Effects of Noise on Aquatic Life I* Vol. 875 *Advances in Experimental Medicine and Biology* (eds Popper, A. N. & Hawkins, A.) 17–26 (2016).

[4] Fewtrell JL, McCauley RD (2012). Impact of air gun noise on the behaviour of marine fish and squid. Mar Pollut Bull.

[5] Guerra A, González A, Rocha F (2004). A review of records of giant squid in the north-eastern Atlantic and severe injuries in Architeuthis dux stranded after acoustic exploration. ICES C. M. CC.

[6] Kight CR, Swaddle JP (2011). How and why environmental noise impacts animals: an integrative, mechanistic review. Ecol Lett.

[7] Lagardère J (1982). Effects of noise on growth and reproduction of Crangon crangon in rearing tanks. Mar. Biol..

[8] Lovell J, Findlay M, Moate R, Yan H (2005). The hearing abilities of the prawn Palaemon serratus. Comp Biochem Physiol. A.

[9] McCauley R.D., Fewtrell J., Duncan A.J., Jenner C., Jenner M-N., Penrose J.D., Prince R.I.T., Adhitya A., Murdoch J., McCabe K. (2000). MARINE SEISMIC SURVEYS— A STUDY OF ENVIRONMENTAL IMPLICATIONS. The APPEA Journal.

[10] Morley E, Jones G, Raddford A (2014). The importance of invertebrates when considering the impacts of anthropogenic noise. Proc Roy Soc B-Biol Sci.

[11] Parry GD, Gason A (2006). The effect of seismic surveys on catch rates of rock lobsters in western Victoria, Australia. Fish Res.

[12] Pearson WH, Skalski JR, Sulkin SD, Malme CI (1994). Effects of seismic energy releases on the survival and development of zoeal-larvae of dungeness crab (Cancer magister). Mar Environ Res..

[13] Tomanek L (2015). Proteomic responses to environmentally induced oxidative stress. J Exp Biol.

[14] Tomanek L, Zuzow MJ (2010). The proteomic response of the mussel congeners Mytilus galloprovincialis and M. trossulus to acute heat stress: implications for thermal tolerance limits and metabolic costs of thermal stress. J Exp Biol.

[15] Varo, I. *et al*. Dietary Effect on the Proteome of the Common Octopus (*Octopus vulgaris*) Paralarvae. **8**, 10.3389/fphys.2017.00309 (2017). 10.3389/fphys.2017.00309 PMC5434110 28567020

[16] Veal, E. *et al*. A 2-Cys peroxiredoxin regulates peroxide-induced oxidation and activation of a stress-activated MAP kinase **15**, 129–139 (2004). 10.1016/j.molcel.2004.06.021 15225554

[17] Carroll, A., Przeslawski, R., Gunning, M. E., Bruce, B. & Duncan, A. J. A critical review of the potential impacts of marine seismic surveys on fish & invertebrates. **114** 9–24 (2017). 10.1016/j.marpolbul.2016.11.038 27931868

[18] Solé M (2013). Does exposure to noise from human activities compromise sensory information from cephalopod statocysts?. Deep-Sea Res. II.

[19] Newson SE (2008). Indicators of the impact of climate change on migratory species. BTO Research Report.

[20] Gasalla MA, Rodrigues AR, Postuma FA (2010). The trophic role of the squid Loligo plei as a keystone species in the South Brazil Bight ecosystem. ICES J Mar Sci.

[21] Pierce, G. J. & Santos, M. B. In *Aquatic predators and their prey* (eds Greenstreet, S. P. R. & Tasker, M. L.) 58–64 (1996).

[22] Rodhouse PG, Nigmatullin CM (1996). Role as consumers. Philos Trans R Soc Lond B Biol Sci.

[23] Santos MB, Clarke MR, Pierce GJ (2001). Assessing the importance of cephalopods in the diets of marine mammals and other top predators: Problems and solutions. Fish Res.

[24] Santos MB (2014). Patterns and trends in diet of long-finned pilot whales (Globicephala melas) based on the analysis of stomachs contents of animals stranded on Northeast Atlantic coasts. Mar Mam Sci.

[25] Hastie, L. C. *et al*. In *Oceanography and Marine Biology: An Annual Review, Vol 47*, *Oceanography and Marine Biology* (eds Gibson, R. N., Atkinson, R. J. A. & Gordon, J. D. M.) 111–190 (2009).

[26] Pierce, G. J. *et al*. Cephalopod Indicators for the MSFD. *Final Report to Defra on Contract No ME5311* (2016).

[27] Pierce GJ, Wang J, Valavanis V (2002). Application of GIS to cephalopod fisheries: Workshop report. Bull Mar Sci.

[28] Budelmann, B. In *The evolutionary biology of hearing* (eds Webster, D. B., Fay, R. A. & Popper, A. N.) 141–155 (Springer, 1992).

[29] Young JZ (1989). The angular-acceleration receptor system of diverse cephalopods. Philos Trans R Soc Lond B Biol Sci.

[30] Budelmann, B. In *Squid as experimental animals* (eds Gilbert, Daniel, L. Adelman, William, J. & Arnold, John, M.) 421–439 (Plenum Press, 1990).

[31] Williamson R, Chrachri A (2007). A model biological neural network: the cephalopod vestibular system. Philos Trans R Soc Lond B Biol Sci.

[32] Radtke R (1983). Chemical and structural characteristics of statoliths from the short-finned squid *Illex illecebrosus*. Mar Biol.

[33] Durholtz M, Kretsinger R, Lipinski M (1999). Unique proteins from the statoliths of Lolliguncula brevis (Cephalopoda: Loliginidae). Comp Biochem Physiol. B.

[34] Bettencourt V, Guerra A (2000). Growth increments and biomineralization process in cephalopod statoliths. J Exp Mar Biol Ecol.

[35] Guerra, A. *Mollusca: Cephalopoda*. Vol. 1 (Consejo Superior de Investigaciones Científicas, 1992).

[36] André, M. *et al*. In *The Effects of Noise on Aquatic Life II Advances in Experimental Medicine and Biology* (eds Popper, Arthur N. & Hawkins, Anthony) 47–55 (Springer New York, 2016).

[37] André M (2011). Low frequency sounds induce massive acoustic trauma in cephalopods. Front Ecol Environ.

[38] Solé M (2013). Ultrastructural damage of *Loligo vulgaris and Illex coindetii* statocysts after Low Frequency Sound Exposure. PLoS ONE.

[39] Solé, M. *et al*. Offshore exposure experiments on cuttlefish indicate received sound pressure and particle motion levels associated with acoustic trauma. *Sci Rep* **7**, 10.1038/srep45899 (2017). 10.1038/srep45899 PMC5381195 28378762

[40] Day, R., McCauley, R., Fitzgibbon, Q., Hartmann, K. & Semmens, J. Assessing the impact of marine seismic surveys on southeast Australian scallop and lobster fisheries, Fisheries Research and Development Corporation. (Government or Industry Research, University of Tasmania, Hobart, 2016).

[41] Hoekstra, K. A., Iwama, G. K., Nichols, C. R., Godin, D. V. & Cheng, K. M. Increased heat shock protein expression after stress in Japanese quail. **2**, 265–272 (1998). 10.3109/10253899809167290 9876257

[42] Payan P, Pontual HD, Bœuf G, Mayer-Gostan N (2004). Endolymph chemistry and otolith growth in fish. C R Palevol.

[43] Vogel Christine, Silva Gustavo Monteiro, Marcotte Edward M. (2011). Protein Expression Regulation under Oxidative Stress. Molecular & Cellular Proteomics.

[44] Nakagawa T (1997). Two modes of auditori hair cell loss following acoustic overstimulation in the avian inner ear. ORL J. Otorhinolaryngol. Relat. Spec..

[45] Ibarz A (2010). Gilthead sea bream liver proteome altered at low temperatures by oxidative stress. Proteomics.

[46] López J, Marina A, Vázquez J, Alvarez G (2002). A proteomic approach to the study of marine mussels Mytilus edulis and M. galloprovincialis. Mar. Biol..

[47] McLean L (2007). Global cooling: Cold acclimation and the expression of soluble proteins in carp skeletal muscle. Proteomics.

[48] Dowd W, Harris B, Cech J, Kültz D (2010). Proteomic and physiological responses of leopard sharks (*Triakis semifasciata*) to salinity change. J Exp Biol.

[49] Smith R, Cash P, Ellefsen S, Nilsson G (2009). Proteomic changes in the crucian carp brain during exposure to anoxia. Proteomics.

[50] Gielens C, Bosman F, Preaux G, Lontie R (1983). Structural studies by limited proteolysis of the haemocyanin of *Sepia officinalis*. Life Chem. Rep..

[51] Havanapan PO, Kanlaya R, Bourchookarn A, Krittanai C, Thongboonkerd V (2009). C-terminal hemocyanin from hemocytes of *Penaeus vannamei* interacts with ERK1/2 and undergoes serine phosphorylation. J Proteome Res..

[52] Schlesinger MJ (1990). Heat shock proteins. J Biol Chem.

[53] Garnier C (1998). Heat-shock protein 90 (hsp90) binds *in vitro* to tubulin dimer and inhibits microtubule formation. Biochem Biophys Res Commun.

[54] Sanchez C, Padilla R, Paciucci R, Zabala JC, Avila J (1994). Binding of Heat-Shock Protein 70 (hsp70) to Tubulin. Arch Biochem Biophys.

[55] Somboonwiwat K, Chaikeeratisak V, Wang H-C, Lo CF, Tassanakajon A (2010). Proteomic analysis of differentially expressed proteins in *Penaeus monodon* hemocytes after Vibrio harveyi infection. Proteome Sci.

[56] Bryk R, Lima C, Erdjument-Bromage H, Tempst P, Nathan C (2002). Metabolic enzymes of mycobacteria linked to antioxidant defense by a thioredoxin-like protein. Science.

[57] Hofmann B, Hecht H, Flohe L (2002). Peroxiredoxins. Biol Chemc.

[58] Covarrubias L, Hernández-García D, Schnabel D, Salas-Vidal E, Castro-Obregón S (2008). Function of reactive oxygen species during animal development: Passive or active?. Dev Biol.

[59] Li L, Nevill G, Forge A (1995). Two modes of hair cell loss from the vestibular sensory epithelia of the guinea pig inner ear. The Journal of comparative neurology.

[60] Cotanche DA (1987). Regeneration of hair cell stereociliary bundles in the chick cochlea following severe acoustic trauma. Hear Res.

[61] Gagnon C (1996). The polyglutamylated lateral chain of alpha-tubulin plays a key role in flagellar motility. J Cell Sci.

[62] Goldman R, Cleland M, Murthy S, Mahammad S, Kuczmarski E (2012). Inroads into the structure and function of intermediate filament networks. J Struct Biol.

[63] Kujawa SG, Liberman MC (2009). Adding insult to injury: Cochlear nerve degeneration after “temporary” noise-induced hearing loss. J Neurosci.

[64] Leonova E, Raphael Y (1997). Organization of cell junctions and cytoskeleton in the reticular lamina in normal and ototoxically damaged organ of Corti. Hear Res.

[65] Raphael Y (2002). Cochlear pathology, sensory cell death and regeneration. Brit Med Bull.

[66] Raphael Y, Altschüler R (1991). Reorganization of cytoskeletal and junctional proteins during cochlear hair cell degeneration. Cell Mot Cytosk.

[67] Säljö A, Bao F, Hamberger A, Haglid K, Hansson H (2001). Exposure to short-lasting impulse noise causes microglial and astroglial cell activation in the adult rat brain. Pathophysiology.

[68] Gonen H (1994). Protein synthesis elongation factor EF-1 alpha is essential for ubiquitin-dependent degradation of certain N alpha-acetylated proteins and may be substituted for by the bacterial elongation factor EF-Tu. Proc Natl Acad Sci USA.

[69] Gonen H, Dickman D, Schwartz AL, Ciechanover A (1996). Protein synthesis elongation factor EF-1 alpha is an isopeptidase essential for ubiquitin-dependent degradation of certain proteolytic substrates. Adv Exp Med Biol.

[70] Chen F, Chang D, Goh M, Klibanov SA, Ljungman M (2000). Role of p53 in cell cycle regulation and apoptosis following exposure to proteasome inhibitors. Cell Growth Differ.

[71] Duttaroy A, Bourbeau D, Wang XL, Wang E (1998). Apoptosis rate can be accelerated or decelerated by overexpression or reduction of the level of elongation factor-1 alpha. Exp Cell Res.

[72] Andrews PLR (2013). The identification and management of pain, suffering and distress in cephalopods, including anaesthesia, analgesia and humane killing. J Exp Mar Biol Ecol.

[73] Fiorito G (2015). Guidelines for the Care and Welfare of Cephalopods in Research -A consensus based on an initiative by CephRes. FELASA and the Boyd Group..

[74] Moltschaniwskyj N (2007). Ethical and welfare considerations when using cephalopods as experimental animals. Rev Fish Biol Fisher.

[75] Solé, M. *et al*. Evidence of Cnidarians sensitivity to sound after exposure to low frequency noise underwater sources. *Sci Rep* **6**, 10.1038/srep37979 (2016). 10.1038/srep37979 PMC5175278 28000727

[76] Solé Marta, Lenoir Marc, Fortuño José-Manuel, van der Schaar Mike, André Michel (2018). A critical period of susceptibility to sound in the sensory cells of cephalopod hatchlings. Biology Open.

[77] Arentz Georgia, Weiland Florian, Oehler Martin K., Hoffmann Peter (2015). State of the art of 2D DIGE. PROTEOMICS - Clinical Applications.

[78] Unlu M, Morgan ME, Minden JS (1997). Difference gel electrophoresis: A single gel method for detecting changes in protein extracts. Electrophoresis.

[79] Alban A (2003). A novel experimental design for comparative two-dimensional gel analysis: two-dimensional difference gel electrophoresis incorporating a pooled internal standard. Proteomics.

[80] Esselens CW (2008). J. Metastasis-associated C4.4A, a GPI-anchored protein cleaved by ADAM10 and ADAM17. Biol. Chem..

[81] Perez-Riverol Y (2019). The PRIDE database and related tools and resources in 2019: improving support for quantification data. (PubMed ID: 30395289). Nucleic Acids Res.

