# Supplementary material for: A proteomic analysis of the statocyst endolymph in common cuttlefish (Sepia officinalis): an assessment of acoustic trauma after exposure to sound
Source: Sci Rep. 2019 Jun 27;9:9340. doi: 10.1038/s41598-019-45646-6 (PMC6597576; doi:10.1038/s41598-019-45646-6)
Supplement: Supplementary file 1 — A proteomic analysis of the statocyst endolymph in common cuttlefish (Sepia officinalis): an assessment of acoustic trauma after exposure to sound [file 41598_2019_45646_MOESM1_ESM.docx]

**A proteomic analysis of the statocyst endolymph in common cuttlefish (*Sepia officinalis*): an assessment of acoustic trauma after exposure to sound**

**M. Solé^1^, M. Monge^2^, M. André^1*^ and C. Quero^3*^**

^1^Laboratory of Applied Bioacoustics, Technical University of Catalonia, Rambla exposició s/n, 08800 Vilanova i la Geltrú, Barcelona, Spain. E-mail: [michel.andre@upc.edu](mailto:michel.andre@upc.edu)

^2^Proteomics Laboratory, Vall d’Hebron Institute of Oncology (VHIO), Edifici Collserola, 08035 Barcelona, Spain

^3^Department of Biological Chemistry and Molecular Modelling, IQAC (CSIC), Jordi Girona 18. 08034 Barcelona, Spain. E-mail: [cqlqob@cid.csic.es](mailto:cqlqob@cid.csic.es)

*Corresponding author

Table S1. Experimental design for CyDye labelling

| **Gel** | **Cye 2** | **Cye 3** | **Cye 5** |
| --- | --- | --- | --- |
| **1** | Pool | Control_1 | T 0h_1 |
| **2** | pool | T 24h_1 | Control_2 |
| **3** | pool | Control_3 | T 0h_2 |
| **4** | pool | T 0h_C | T 24h_2 |
| **5** | pool | T 0h_4 | T 24h_3 |
| **6** | pool | T 24h_4 | Control_4 |

Controls are endolymph samples from untreated animals, T_0h and T_24H correspond to endolymph samples from sound exposed animals 0h or 24 h after treatment . Pooled samples are mixtures of the two of the three samples for internal standard.

Table S2 *Sepia officinalis* proteins with significant differential expression between different treatments (Control (C), 0h (T0) and 24h treated (T24)) and which were increased (+) or decreased (-) 1.4-fold, are indicated in bold (p<0.05).

|  | **#** | **Identification** | **Increase/decrease** | | | **C/T0** | | **C/T24** | | **T0/T24** | |
| --- | --- | --- | --- | --- | --- | --- | --- | --- | --- | --- | --- |
|  |  |  | **C/T0** | **C/T24** | **T0/T24** | **Fold** | **Anova (p)** | **Fold** | **Anova (p)** | **Fold** | **Anova (p)** |
| **Hemocyanin** | 75 | hemocyanin subunit 2 [*Sepia officinalis*] | = | = | - | -1.48 | 0.281 | 1.57 | 0.076 | **2.32** | **0.037** |
|  | 114 | hemocyanin subunit 2 [*Sepia officinalis*] | = | - | = | -1.22 | 0.701 | **1.85** | **0.037** | 2.27 | 0.063 |
|  | 280 | hemocyanin subunit 2 [*Sepia officinalis*] | + | = | - | **-1.69** | **0.017** | 1.21 | 0.112 | **2.46** | **0.006** |
|  | 359 | hemocyanin subunit 2 [*Sepia officinalis*] | = | - | = | -1.65 | 0.971 | 1.49 | 0.047 | 2.05 | 0.556 |
|  | 626 | hemocyanin subunit 2 [*Sepia officinalis*] | = | + | = | -1.24 | 0.556 | **-1.53** | **0.029** | -1.23 | 0.248 |
|  | 986 | hemocyanin, units G and H | = | - | = | 0.468 | 0.110 | **1.46** | **0.006** | 1.15 | 0.566 |
|  | 456 | hemocyanin subunit 2 [*Sepia officinalis*] | = | = | - | -1.25 | 0.193 | 1.29 | 0.102 | **1.62** | **0.016** |
|  | 1357 | hemocyanin subunit 2 [*Sepia officinalis*] | = | - | = | 1.61 | 0.111 | **1.59** | **0.038** | 1.02 | 0.738 |
| **Elongation** | 488 | elongation factor-1 alpha [*Quilaphoetosus monachus*] | = | = | + | 1.22 | 0.303 | -2.38 | 0.094 | **-2.90** | **0.048** |
|  | 620 | elongation factor 1-alpha *[Xyleborus biuncus*] | = | - | = | 1.10 | 0.308 | **1.47** | **0.025** | 1.34 | 0.097 |
|  | 869 | elongation factor 1-a [*Hunterella nodulosa*] | = | = | + | 1.30 | 0.218 | -1.80 | 0.065 | **-2.26** | **0.024** |
|  | 1251 | elongation factor-1 alpha [*Chloreuptychia arnaca*] | = | + | = | -1.16 | 0.649 | **-2.57** | **0.045** | -2.21 | 0.094 |
|  | 964 | elongation factor-1 alpha [*Maculinea arion*] | - | = | = | **1.5** | **0.026** | 1.23 | 0.114 | -1.22 | 0.286 |
| **Others** | 216 | heat shock protein 90 [*Dendronephthya klunzingeri*] | = | - | = | 1.22 | 0.304 | **1.44** | **0.009** | 1.18 | 0.705 |
|  | 456 | tubulin alpha chain | = | = | - | -1.25 | 0.193 | 1.29 | 0.102 | **1.62** | **0.016** |
|  | 899 | thioredoxin peroxidase BgTPx [*Biomphalaria glabrata*] | - | = | = | **1.60** | **0.004** | 1.22 | 0.229 | -1.31 | 0.189 |
|  | 1253 | tubulin alpha chain | = | - | = | 1.37 | 0.204 | **1.45** | **0.013** | 1.06 | 0.899 |
|  | 1260 | proteasome alpha subunit | = | + | + | -1.19 | 0.438 | **-2.97** | **0.019** | **2.50** | **0.015** |
|  | 1261 | glyceraldehyde-3-phosphate dehydrogenase 1 | = | + | = | -1.29 | 0.584 | **-1.39** | **0.041** | -1.08 | 0.598 |
|  | 1349 | 70 kDa neurofilament protein | = | - | = | 1.23 | 0.241 | **1.64** | **0.025** | 1.33 | 0.252 |
|  | 1357 | intermediate filament protein [*Nototodarus sloanii*] | = | - | = | 1.61 | 0.111 | **1.59** | **0.038** | 1.02 | 0.738 |


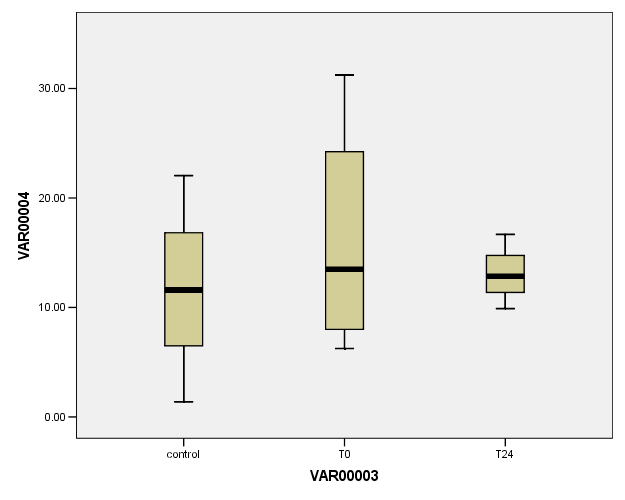
Fig S1. Protein content (mean $\pm$ SD) per statocyst in control (N= 24), 0h after exposure (N=32) and 24h after exposure (N=24). No significant differences were found in the protein concentration between treated and untreated individuals (Anova, p<0.05)

Protein content (µg/

Fig S2. Black and white scan of two-dimensional electrophoresis map of Sepia officinalis endolymph stained with Flamingo fluorescent gel stain (Bio-Rad). Soluble proteins were separated on linear IPG-strips (pH 3–10) followed by 12.5% SDS-PAGE. Molecular mass markers are indicated on the right in KDa


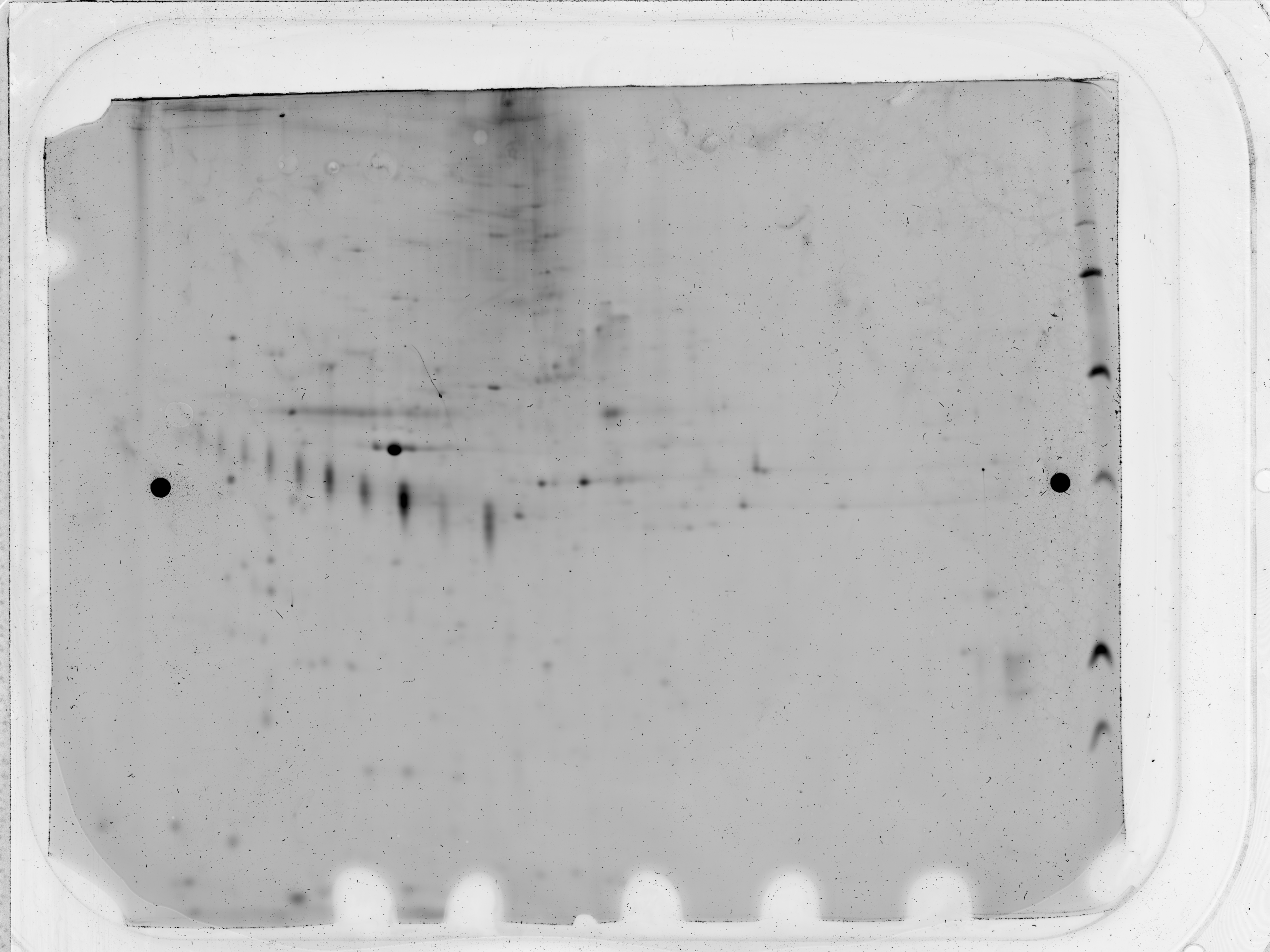


50

75

100

150

250

37

25

20

MW
